# Methylene Blue and Rhodamine B Dyes’ Efficient Removal Using Biocarbons Developed from Waste

**DOI:** 10.3390/molecules29174022

**Published:** 2024-08-25

**Authors:** Robert Wolski, Aleksandra Bazan-Wozniak, Agnieszka Nosal-Wiercińska, Robert Pietrzak

**Affiliations:** 1 Department of Applied Chemistry, Faculty of Chemistry, Adam Mickiewicz University in Poznań, Uniwersytetu Poznańskiego 8, 61-614 Poznań, Poland; robert.wolski@amu.edu.pl; 2 Department of Analytical Chemistry, Institute of Chemical Sciences, Faculty of Chemistry, Maria Curie-Skłodowska University in Lublin, Maria Curie-Skłodowska Sq, 3, 20-031 Lublin, Poland; agnieszka.nosal-wiercinska@mail.umcs.pl

**Keywords:** biomass, biocarbon, methylene blue, rhodamine B, isotherm study, kinetic model, thermodynamic study

## Abstract

The preparation of biocarbons from cellulose fibres utilised in the production of baby nappy mats (sourced from Feniks Recycling company, Poland) for the removal of methylene blue and rhodamine B dyes has been documented. A Brunauer, Emmett and Teller analysis revealed a surface area within the range of 384 to 450 m^2^/g. The objective of this study was to investigate the removal efficiency of dyes from aqueous solutions by biocarbons, with a particular focus on the influence of various parameters, including pH, dye concentration, adsorbent dosage, shaking speed, contact time, and temperature. The maximum adsorption capacity of the dyes onto the biocarbons was found to be 85 mg/g for methylene blue and 48 mg/g for rhodamine B, respectively. The Langmuir equation proved to be the most suitable for interpreting the sorption of organic dyes. The adsorption process was found to exhibit a chemisorption mechanism, effectively mirroring the pseudo-second-order kinetics. Furthermore, the adsorption of dyes was observed to be endothermic (the enthalpy change was positive, 9.1–62.6 kJ/mol) and spontaneous under the tested operating conditions. The findings of this study indicate that biocarbons represent a cost-effective option for the removal of methylene blue and rhodamine B. The adsorption method was observed to be an effective and straightforward approach for the removal of these dyes. The results of the Boehm titration analysis and zero charge point value indicated that the synthesised biomaterials exhibited a slightly basic surface character.

## 1. Introduction

The continuous industrial development and population growth observed in recent years have resulted in an increase in the pollution emitted by various industries [1,2,3,4,5]. In response to these developments, the standards for the amount of pollutant emissions permitted are undergoing a process of revision. These changes have forced industrial plants to adapt their pollution removal methods to the new standards. Consequently, effective solutions for reducing the amount of pollution are being sought, and one method is the use of adsorption processes [6,7]. The most commonly employed adsorbents include silica gels, zeolites, mesoporous silica materials and activated carbons [8,9,10].

Activated carbon is a highly sought-after material due to its optimally developed specific surface area and porous structure. It is also renowned for its impressive mechanical and chemical strength [11]. The adsorption capacity of activated carbon is often attributed to the presence of various functional groups in its structure [12]. A range of organic materials containing significant quantities of elemental carbon in their composition can be employed as precursors for the production of activated carbons. These include lignin, peat, wood and fruit seeds [13,14,15]. The processing of the precursors allows for the production of activated carbons with the desired adsorption properties. Consequently, activated carbon is employed in the removal of organic and inorganic contaminants from both liquid and gas phases. Additionally, it is utilised in decontamination processes and as a catalyst carrier or catalyst [16,17,18].

The search for savings in the production of activated carbons has led to the use of waste materials as precursors. Increasingly, industry is utilising waste materials as a source of raw materials for the production of activated carbons [19]. The carbon precursor plays a pivotal role not only in terms of economics, but also in terms of the physicochemical properties and adsorption capacity of the carbon material. The selection of an appropriate precursor can significantly influence the final properties of the activated carbon [20]. In addition to the economic and environmental benefits, the use of precursors derived from waste materials facilitates sustainable development, contributing to waste reduction and reducing pressure on natural resources [21]. To illustrate, cellulose fibre-activated carbons can exhibit a high specific surface area, which enables them to effectively adsorb pollutants and chemicals. Furthermore, the production of activated carbon from cellulose fibres utilises renewable raw materials, such as wood and plant waste, thereby rendering the process more sustainable than the utilisation of non-renewable raw materials. The biodegradability of cellulose fibres renders products derived from them more environmentally friendly. Activated carbons produced from cellulosic fibres are chemically stable and retain their adsorption properties in diverse environmental conditions. Furthermore, cellulose fibre-based activated carbons can be modified according to the requirements of the intended application, thereby enabling them to be tailored to specific applications, such as the selective removal of particular contaminants [22,23,24].

Textile dyes such as rhodamine B and methylene blue are widely used in the textile industry due to their vibrant colours and effective dyeing properties. However, their release into water bodies presents several environmental and health concerns. Dyes such as methylene blue and rhodamine B are recognised for their toxicity to aquatic microorganisms, which can disrupt the equilibrium of the entire aquatic ecosystem. Their presence can have a detrimental impact on the development, functionality and survival of organisms that are pivotal to maintaining the health of the ecosystem. Furthermore, these dyes have the capacity to accumulate in living organisms. This implies that they can accumulate in the bodies of fish, aquatic plants and other organisms [25,26,27]. It is estimated that in excess of 700,000 tonnes of industrial dyes are utilised on an annual basis, with in excess of 10% of this quantity entering wastewater systems [28].

The objective of this study was to evaluate the sorption capacity of activated carbons derived from cellulose fibres utilised in the production of baby nappy mats in the adsorption of aqueous solutions of organic dyes, namely methylene blue and Rhodamine B. The distinctive aspect of this research is the utilisation of cellulose fibres, which are employed in the production of baby nappy mats, as a precursor. To the best of our knowledge, this waste has not been employed in the synthesis of activated carbons. The combination of this precursor with a fast and low-cost method of activating bio-waste with carbon dioxide represents another novel aspect of this study. The structural and textural characteristics of the presented activated carbons were determined using various techniques, including Brunauer–Emmett–Teller (BET) analysis, elemental analysis and Boehm titration. Moreover, the kinetics, thermodynamics and isotherms associated with the adsorption process of the organic dyes under investigation have been analysed and presented in this research work.

## 2. Results and Discussion

### 2.1. Characterisation of the Biocarbons

**Figure 1:**
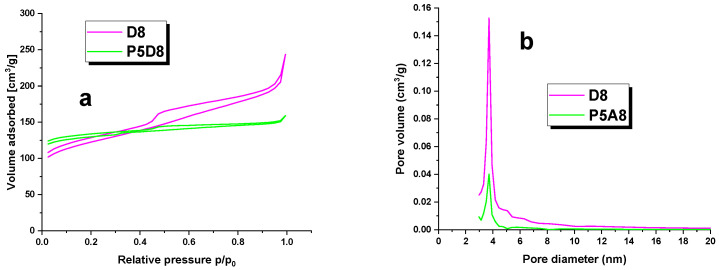
Nitrogen adsorption—desorption isotherms (**a**) and pore-size distribution curves (**b**) of biocarbons.

**Figure 2:**
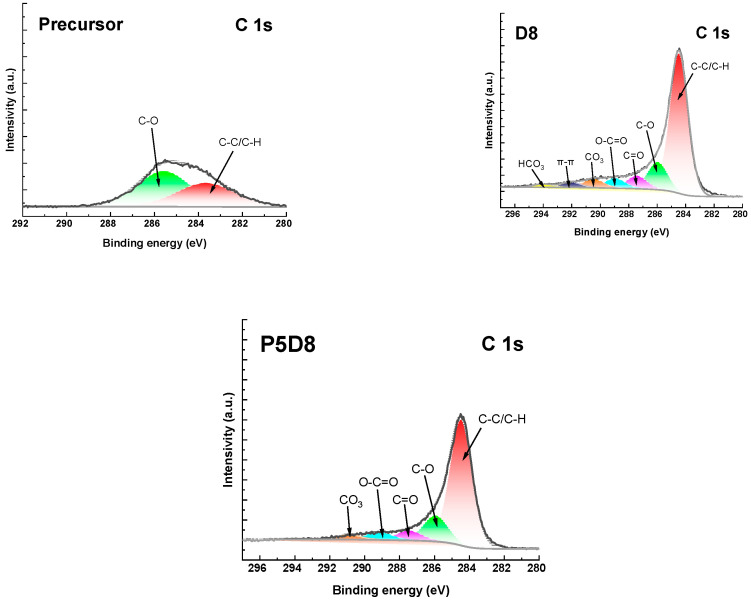
XPS C1s spectra of precursors and biocarbons.

The surface area of the biocarbons, the total pore and micropore volume, and the average pore diameter of the D8 and P5D8 samples were calculated using the nitrogen adsorption/desorption isotherms illustrated in Figure 1. The textural parameters are presented in tabular form in Table 1. The adsorbents were found to exhibit a relatively low specific surface area. It is conceivable that the temperatures employed during carbonisation and activation were inadequate to effectively develop the specific surface area. An additional possibility is that the temperature differential between the two processes was insufficient. The surface area of the samples obtained was that of the D8 and P5D8. The compiled values of specific surface area clearly indicate that the two-step physical activation process allows the production of an adsorbent with a microporous texture character, as also confirmed in Figure 1 and Figure 2. Table 1 shows that the pore diameters of the four samples analysed were within a narrow range, from 2.57 to 3.36 nm. The average pore size for the activated carbons provides evidence for the formation of mesopores with smaller sizes. Furthermore, the specific surface area of the samples correlates with their iodine number, as both parameters reflect the low degree of porosity of the carbonaceous material.

A comparative analysis of the specific surface area of the samples presented in Table 1 and the results documented in the literature for carbon adsorbents derived from waste materials through the activation process with carbon(IV) oxide reveal that the surface area of samples D8 and P5D8 is comparable or even higher than those reported in other studies [21,29,30]. The findings of this study indicate that rinsing the samples with an acid solution following the activation process may result in an enhanced specific surface area [21]. Consequently, the forthcoming research will entail the washing of the coals obtained from this type of precursor with a mixture of acids, with a view to determining their textural parameters.

The surface area of the adsorbent exerts a significant influence on the adsorption process, particularly in the context of the presence of oxygen-containing surface groups. The Boehm method was employed to ascertain the quantity of acidic and basic oxygen-containing functional groups, thereby enabling an investigation of the acid–base characteristics of the surface of the samples under examination. The results of these measurements are presented in Table 2 for reference. The data collected indicate that the proposed method of obtaining activated carbons from this type of precursor results in the production of adsorbents with a slightly alkaline surface character. The content of oxygen functional groups of an acidic nature falls within a narrow range of 0.46 to 0.48 mmol/g, while the basic groups oscillate between 0.67 and 1.06 mmol/g. The data analysis revealed that direct activation of the precursor with carbon(IV) oxide resulted in a slight increase in both acidic and basic functional groups. The pH_pzc_ values indicate that samples D8 (9.8) and P5D8 (8.1) exhibited a slight predominance of basic functional groups.

The results of the elemental analysis (C, H, N and S) of the biocarbons are presented in Table 3. The precursor is distinguished by a relatively low elemental carbon content, amounting to 42.13 wt. %. Accordingly, the initial material was subjected to physical and direct activation with CO_2_ in order to enhance the degree of carbonisation and ordering of the carbon structure. As can be observed, the biocarbons exhibit a markedly elevated proportion of elemental carbon. An increase in C^daf^ is associated with a concomitant decrease in hydrogen and oxygen. The reduction in H^daf^ and O^daf^ is attributed to the influence of elevated temperatures, which facilitate the disruption of less stable chemical bonds. The observed increase in nitrogen and sulphur is plausibly attributable to the presence of thermally stable heterocyclic linkages of these elements in the precursor. It is noteworthy that both the precursor and samples D5 and P5D8 exhibit a relatively low mineral content.

**Figure 3:**
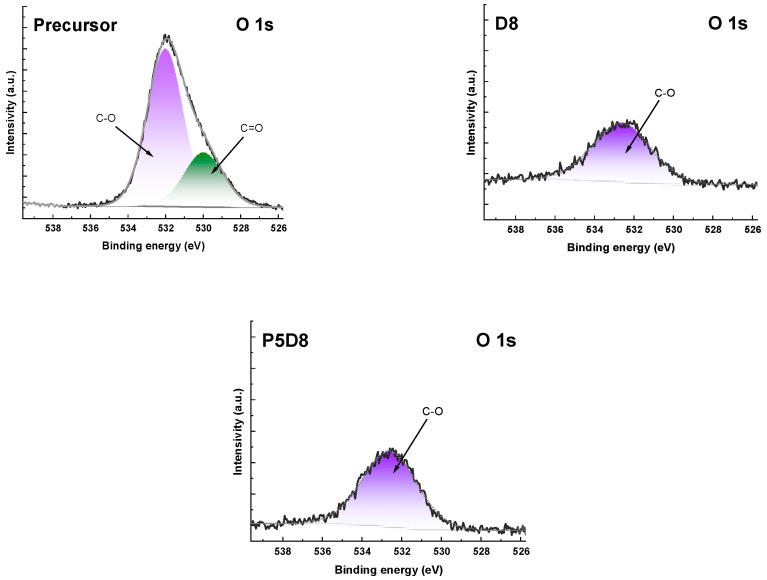
XPS O1s spectra of precursors and biocarbons.

The high-resolution XPS (X-ray photoelectron spectroscopy) spectra of C1s and O1s are presented in Figure 2 and Figure 3, respectively. The C1s spectra could be resolved into two to seven peaks, which were assigned, among others, to aromatic C-C/C-H, aromatic C-O, ketone C=O, carboxylic O-C=O and the π-π bond. The O1s spectra could be resolved into two peaks, which were related to C-O groups and C=O groups, as demonstrated in Figure 3 [31].

### 2.2. Adsorption Study

**Figure 4:**
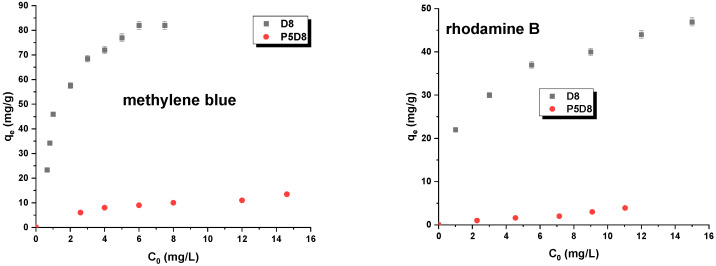
Isotherms of methylene blue and rhodamine B adsorption on D8 and P5D8 samples. Experimental conditions: C_0_ = 10–40 mg/L, mass = 0.020 g, adsorption time = 24 h, shaking speed = 200 rpm/min, temperature = 295 ± 1 K.

**Figure 5:**
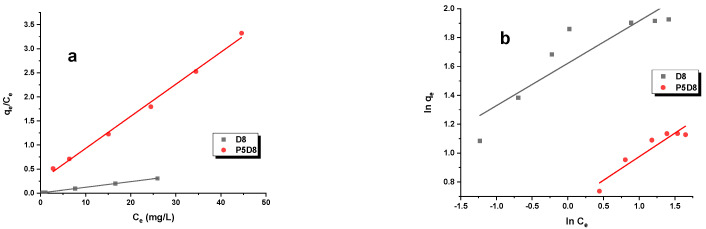

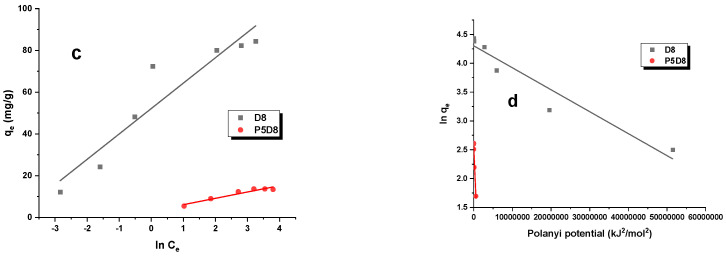
(**a**) Langmuir, (**b**) Freundlich, (**c**) Temkin and (**d**) Dubinin–Radushkevich isotherm models for methylene blue adsorption on biocarbons. Experimental conditions: C_0_ = 10–40 mg/L, mass = 0.020 g, adsorption time = 24 h, shaking speed = 200 rpm/min, temperature = 295 ± 1 K.

**Figure 6:**
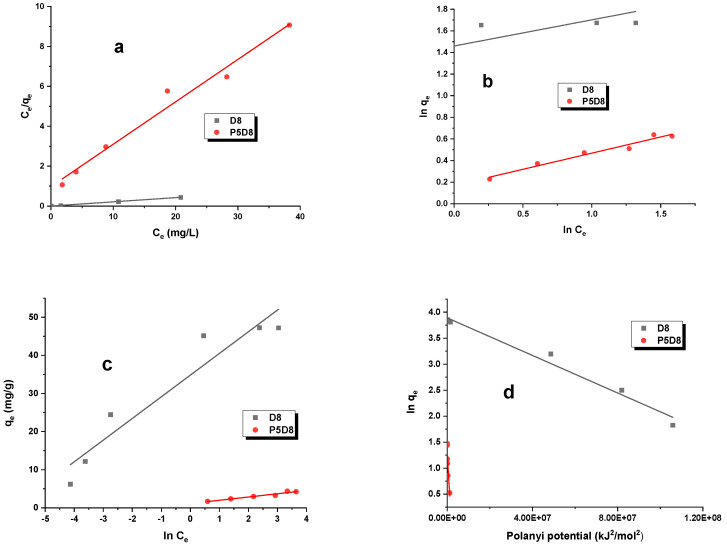
(**a**) Langmuir, (**b**) Freundlich, (**c**) Temkin and (**d**) Dubinin–Radushkevich isotherm models for rhodamine B adsorption on biocarbons. Experimental conditions: C_0_ = 10–40 mg/L, mass = 0.020 g, adsorption time = 24 h, shaking speed = 200 rpm/min, temperature = 295 ± 1 K.

The adsorption of organic compounds on biocarbon can be described by a number of different adsorption isotherm models, including the Langmuir, Freundlich, Temkin and Dubinin–Radushkevich models. Table 4 presents the parameters of these isotherms, which were obtained through linear regression analysis, as well as the experimentally determined sorption capacities (Table 3, Figure 4). The straight lines representing these models are illustrated in Figure 5 and Figure 6. The data presented in Table 4 and Figure 4 clearly demonstrate that sample D8, obtained by activating the cellulose fibres utilised in the production of nappy mats, exhibited a markedly enhanced efficiency in the removal of the adsorbates under investigation. The carbon demonstrated the capacity to adsorb 84 mg/g of methylene blue and 47 mg/g of rhodamine B. In comparison, the experimental sorption capacities for sample P5D8 were observed to be below 15 mg/g. The results of the sorption capacity analysis indicate that the two-step physical activation of this specific precursor does not result in highly effective adsorbents for the removal of the pollutants under investigation. From an economic standpoint, this is a significant consideration, as an additional process step, such as pyrolysis, introduces additional costs in the synthesis of this type of material. Consequently, further research on this precursor should encompass the preparation of bio-adsorbents through direct activation with carbon dioxide, utilising varying temperatures and process times to obtain biomaterials with the most optimal textural and sorption characteristics.

The experimental sorption capacities obtained are correlated with the size of the specific surface area of the synthesised materials. Furthermore, the methylene blue molecule has a smaller kinetic diameter, which facilitates its incorporation into the active sites of samples D8 and P5D8 in comparison to the rhodamine B molecule. Additionally, sample D8 had a greater number of alkali groups, which could interact with cationic impurities such as methylene blue and rhodamine B.

An isotherm is defined as a mathematical equation that describes the relationship between the quantity of the adsorbate that is adsorbed and its equilibrium concentration at a constant temperature. In this study, the parameters of these isotherms were determined based on the analysis of experimental data. The R^2^ and AdjR^2^ parameters were employed to identify the optimal isotherm model that best aligns with the experimental data.

As evidenced in Table 4, the Langmuir model was identified as the optimal isotherm model. The adsorption isotherms illustrated in Figure 5 and Figure 6 also indicate that the investigated process followed this model. Consequently, it can be postulated that the study involves monolayer adsorption on the surface of biocarbon materials, which possess a limited number of identical adsorption sites. The maximum theoretical sorption capacities determined for activated carbons against methylene blue and rhodamine B range from 5 to 85 mg/g.

In the case of the Freundlich isotherm, where adsorption occurs on a heterogeneous adsorbent surface, the stronger binding sites are occupied first, and the binding strength decreases as the degree of site occupancy increases. This phenomenon can be quantified by a parameter, 1/n, which is defined as follows: For the biocarbons, the value of this parameter was less than one, indicating that the adsorption of the dyes was energetically favourable. The highest value of the constant K_F_ for adsorbent D8 (41.869 mg/g(L/mg)^1/n^, methylene blue) suggests the strongest interaction between dye and adsorbent for this sample. 

In the Dubinin–Radushkevich isotherm, the values of the parameter E are useful for determining the type of adsorption. Energy values within the range of 1.0 to 8.0 kJ/mol and 8.0 to 16.0 kJ/mol indicate the presence of physical and chemical adsorption, respectively. As evidenced by the data presented in Table 4, the adsorption of methylene blue and rhodamine B on samples D8 and P5D8 was predominantly governed by physical forces, including hydrogen bonds and van der Waals interactions. It should be noted, however, that the calculated R^2^ and AdjR^2^ coefficients for the three remaining models (Freundlich, Temkin and Dubinin–Radushkevich) indicated that they did not exert a significant influence on the determination of the mechanism of the guest–host reaction.

A further objective of the research was to compare the sorption capacities obtained with those reported in previously published studies. This is essential for evaluating the potential of the synthesised adsorbent in terms of pollutant removal. Consequently, a comparison was conducted between the results obtained in this article and those previously published. The collected data are summarised in Table 5. By analysing the maximum sorption capacities for methylene blue and rhodamine B shown in Table 5, it can be concluded that the D8 biocarbon exhibited comparable or lower sorption capacities. It is plausible that the sorption capacities observed for samples D8 and P5D8 were significantly influenced by the degree of development of the porous structure and the specific surface area. Consequently, this may have resulted in a restricted number of active sites for the adsorbate molecules.

**Figure 7:**
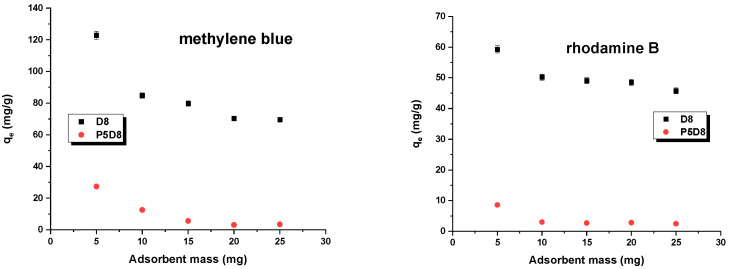
Effect of sorbent mass on adsorption. Experimental conditions: C_0_ (methylene blue) =  30 mg/L, C_0_ (rhodamine B) = 25 mg/L, adsorption time = 24 h, shaking speed = 200 rpm/min, temperature = 295 ± 1 K.

**Figure 8:**
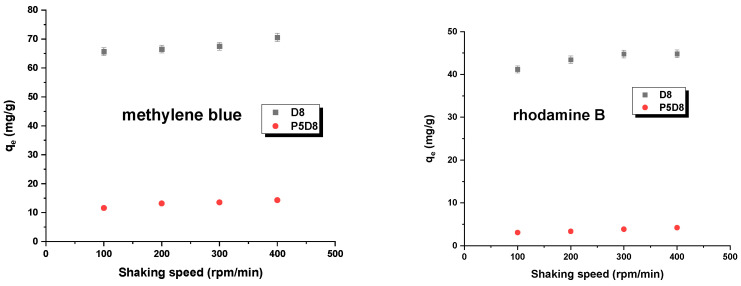
Effect of shaking speed on adsorption. Experimental conditions: C_0_ (methylene blue) =  30 mg/L, C_0_ (rhodamine B) = 25 mg/L, mass = 0.020 g, adsorption time = 24 h, temperature = 295 ± 1 K.

In this study, the effects of adsorbent mass and shaking rate on the sorption capacity of the adsorbents used were investigated by varying the aforementioned parameters, mass (0.005–0.03 g) and shaking speed (100–300 rpm/min), while keeping the dye concentration, temperature and contact time constant. The results are summarised in Figure 7 and Figure 8. As shown in Figure 7, increasing the biocarbon mass led to a reduction in the amount of dye adsorbed. This effect is associated with the decreasing ratio of dye to adsorbent mass, resulting in the lower efficiency of the active sites on the adsorbent surface. The results presented in Figure 8 may be attributed to the observation that an increase in mixing speed facilitated the diffusion of dyes into the pores of the biochars. This also indicated that a shaking speed within the range of 200–300 rpm/min was adequate to guarantee uncomplicated access to the maximum adsorption sites within the pores of the adsorbents, thereby facilitating the adsorption of methylene blue/rhodamine B. For the sake of convenience, a shaking speed of 200 rpm was selected for the tests [37,38].

**Figure 9:**
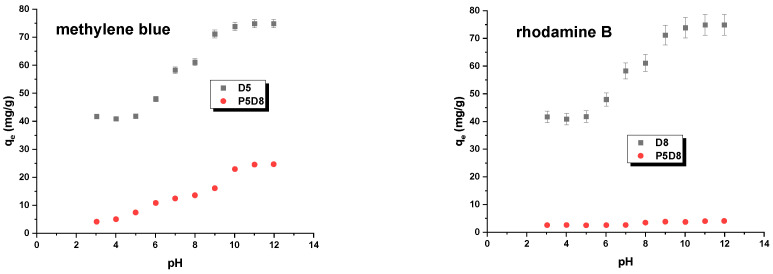
Effect of pH on methylene blue and rhodamine B adsorption on biocarbons. Experimental conditions: C_0_ (methylene blue) = 30 mg/L, C_0_ (rhodamine B) = 25 mg/L, mass = 0.020 g, adsorption time = 24 h, shaking speed = 200 rpm/min, temperature = 295 ± 1 K.

The pH of the environment in which the adsorption process occurs can also influence the efficiency with which toxic compounds and contaminants are removed. The pH of the solution is considered to be one of the most relevant parameters, as it has the potential to modify the charge of both the adsorbate and the adsorbent during the course of testing. Figure 9 illustrates the impact of pH on the sorption capacities obtained. The adsorption of organic pollutants is enhanced as the pH of the system increases. This parameter exerts a more pronounced influence on sample D8. It can be posited that pH exerts a more pronounced influence on the adsorption of methylene blue, as evidenced by the observation of a greater increase in sorption capacity with rising system pH for this dye. This phenomenon can be attributed to the electrostatic interactions between the negatively charged surface of the D8 and P5D8 samples and the methylene blue/rhodamine B molecules [39]. Furthermore, a pH value of the solution exceeding pH_pzc_ signifies that the adsorbent surface is negatively charged, thereby facilitating interaction with positively charged methylene blue or rhodamine B. To guarantee that the surface of the biocarbon is negatively charged, the pH of the system should be higher than the pH_pzc_ value of the biocarbon (Table 2).

**Figure 10:**
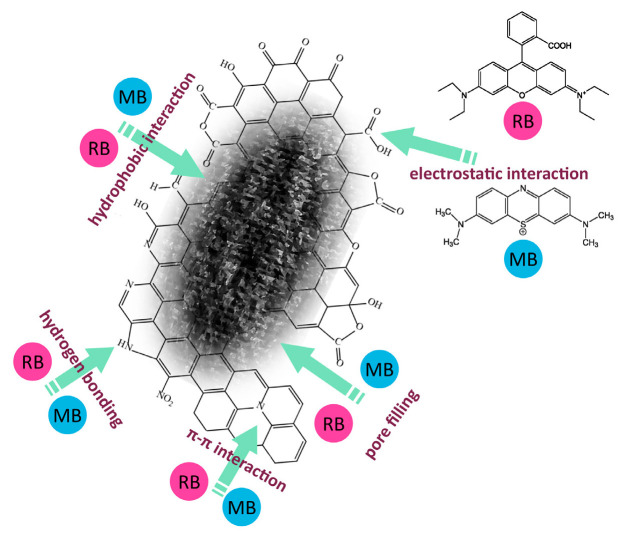
Interactions of methylene blue (MB)/rhodamine B (RB) with the surface of biocarbon.

Figure 10 illustrates the potential interactions between methylene blue and rhodamine B molecules and the surface of samples D8 and P5D8. The adsorption process can occur via a number of different mechanisms, including electrostatic attraction, π-π interactions between the aromatic rings present in the biocarbon and the methylene blue/rhodamine B molecules, the formation of hydrogen bonds, or interactions between the aforementioned aromatic ring and a heteroatom that possesses lone electron pairs.

**Figure 11:**
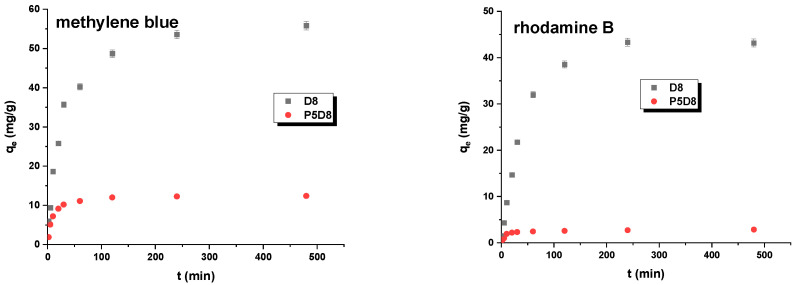
Effect of contact time on the adsorption of organic dyes on biocarbons.

In order to ascertain the requisite equilibrium adsorption time, the adsorption of methylene blue and rhodamine B on the D8 and P5D8 samples was examined as a function of contact time. The resulting data are presented in Figure 11. As observed, the adsorption kinetics of methylene blue and rhodamine B were rapid at the outset of the process, with equilibrium being reached after approximately 100–250 min. The rapid adsorption process at the outset of the contact period can be attributed to the high number of active sites on the biocarbon material surface. However, the slower rate of dye adsorption in the subsequent stages was likely due to the slow diffusion of dye molecules from the solution to the surface and into the pores of the adsorbent [40].

In order to elucidate the mechanism and rate of methylene blue/rhodamine B adsorption on biocarbons, the experimental kinetic data were modelled using two kinetic models: the pseudo-first-order and pseudo-second-order models. The principal parameters of these models are presented in Table 6.

**Figure 12:**
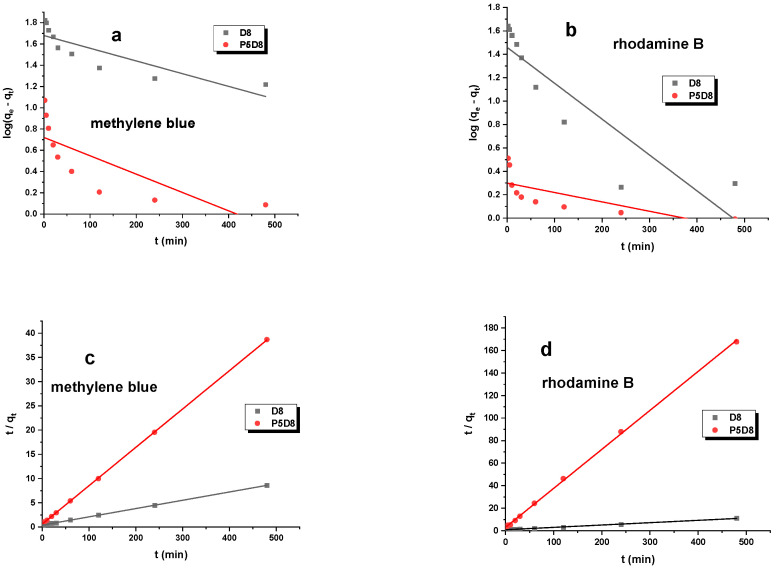

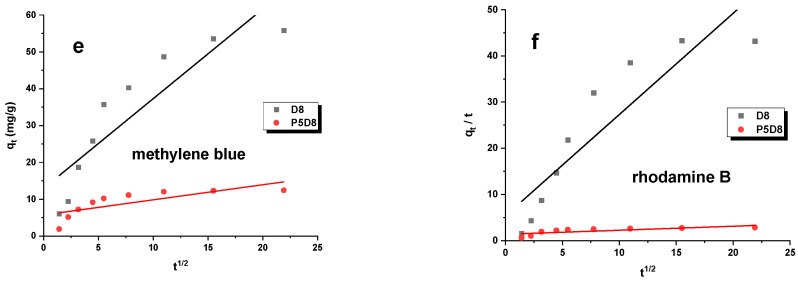
Pseudo-first-order (**a**,**b**), pseudo-second-order (**c**,**d**) and intraparticle diffusion (**e**,**f**) kinetic plots for adsorption of organic dyes on biocarbons. Experimental conditions: C_0_ (methylene blue) = 30 mg/L, C_0_ (rhodamine B) = 25 mg/L, mass = 0.020 g, adsorption time = 24 h, shaking speed = 200 rpm/min, temperature = 295 ± 1 K.

The results presented in Table 6 and Figure 12 demonstrate that the q_e,cal_ value calculated for the pseudo-second-order model exhibited a closer alignment with the actual sorption capacity determined through experimentation. This indicated that the pseudo-second-order kinetics exhibited a superior fitting effect. In contrast, the k_2_ values indicated that the adsorption rate was higher for sample P5D8 than for D8, irrespective of the dye employed. Additionally, the size of the dye molecules can influence the adsorption rate. In the case of rhodamine B, whose structure is larger in comparison to methylene blue, it is possible that the molecules were unable to penetrate deeply into the pores of the adsorbent, resulting in the rapid occupation of the available active sites of the biocarbon. Nevertheless, the outcomes of the k_2_ constant, as illustrated in Table 6, indicate that the pivotal factor in the adsorption process is not the size of the dye, but rather the chemical composition and surface area of the biocarbon.

The data presented in Table 6 suggest that the chemical interaction between methylene blue and rhodamine B and the D8/P5D8 sample exerts a significant influence on the adsorption rate. It can thus be concluded that chemisorption represents the most significant rate-limiting step. The accessibility of biocarbon active sites exerted a pivotal influence on the removal rate of the pollutants under examination. These findings indicate that the reaction was predominantly based on chemisorption [41].

The adsorption of methylene blue and rhodamine B on porous materials was investigated at varying temperatures to ascertain the thermodynamic parameters governing the adsorption process. The results demonstrate that the process temperature had a negligible impact on the sorption capacities attained (Table 7). Changes in temperature affect the equilibrium capacity of the biocarbon for a particular dye, as the diffusion rate of methylene blue/rhodamine B molecules is a temperature-controlled process. Consequently, increasing the process temperature permitted adsorbate molecules to diffuse rapidly towards the outer boundary layer and inner pores of samples D8 and P5D8, as viscous forces in solution provided less resistance. The spontaneity of the adsorption process was indicated by the negative value of ΔG^0^. Adsorption was more likely to occur spontaneously at elevated temperatures, as indicated by the positive values of ΔH^0^ and ΔS^0^ [42,43]. Furthermore, when the enthalpy change (∆H^0^) is less than 80 kJ/mol, the process is classified as physical adsorption. Conversely, when the enthalpy change is between 80 and 200 kJ/mol, chemisorption occurs. In contrast to physical adsorption, which is primarily governed by van der Waals interactions, chemisorption entails the formation of hydrogen, covalent and ionic bonds. In light of the aforementioned criteria, it can be posited that physical adsorption was the prevailing phenomenon observed in the samples under examination. Nevertheless, in light of the comprehensive body of evidence accumulated through these studies, it seems plausible to suggest that both processes may have occurred concurrently. Consequently, further research on this topic is recommended.

Similar outcomes, namely negative Gibbs free energy values and positive enthalpy and entropy values, were observed in our preceding research on the adsorption of methylene blue on adsorbents obtained through the activation of fermentation residues derived from corn (*Zea mays*) stalks and leaves. In this instance, the results for the parameter ΔH^0^ ranged from 9.53 to 10.85 kJ/mol, while the results for ΔS^0^ ranged from 40.11 to 63.61 kJ/mol [44]. The commercial coal CWZ-22 from GRYFSKAND (Hajnówka, Poland) demonstrated positive enthalpy values (below 27 kJ/mol) when tested for the removal efficiency of aqueous rhodamine B solutions [45]. These results are comparable to those obtained in our work. However, the entropy values for commercial carbon exceeded 100 kJ/mol, which is significantly higher than those observed for the D8 and P5D8 samples. This may suggest that the CWZ-22 carbon has a significantly higher selectivity towards the removed dye than samples D8 and P5D8.

### 2.3. Desorption Study

The desorption process, particularly in the context of activated carbon, is of significant importance in a number of industrial sectors, most notably in the purification of water and air. It is crucial for the regeneration of activated carbon, allowing for its repeated utilisation. The efficiency of desorption is contingent upon the chemical properties of both the adsorbate and the eluent employed.

The present study describes a desorption process carried out on D8 carbon, the results of which are presented in Table 8. Three eluents were employed for the purpose of desorption. The eluents employed were 0.1 M HCl, 0.1 M NaOH and distilled water. The highest desorption efficiency was observed with 0.1 M HCl, and the desorption efficiency decreased in the following order: The results demonstrated that HCl exhibited the highest desorption efficiency, followed by H_2_O and NaOH. This phenomenon can be attributed to the displacement of dye molecules from their binding sites in an acidic environment. This study confirmed the regeneration potential of biocarbon. However, to enhance the efficiency of the dye desorption process, it is recommended to consider alternative eluents that could potentially improve the overall efficiency.

## 3. Materials and Methods

### 3.1. Materials

The initial material employed in the research presented in this work was cellulose fibres, which were utilised in the production of baby nappy mats (sourced from Feniks Recycling company, Grodzisk Mazowiecki, Poland). The precursor was cut into strips measuring 12 cm by 2 cm. 

Methylene blue (IUPAC name: 3,7-bis(dimethylamino)-phenothiazin-5-ium chloride, chemical formula: C_16_H_18_ClN_3_S and molecular weight: 319.85 g/mol) and rhodamine B (IUPAC name: 9-(2-carboxyphenyl)-6-(diethylamino)-N,N-diethyl-3H-xanthen-3-iminium chloride, chemical formula: C_28_H_31_ClN_2_O_3_ and molecular weight: 479.02 g/mol) were purchased from Merck (Darmstadt, Germany). Hydrochloric acid and sodium hydroxide and the other chemicals were purchased from Sigma-Aldrich (Burlington, MA, USA) and were of analytical grade. The carbonisation process was carried out in a nitrogen atmosphere (technical nitrogen 4.0, Linde Gaz Poland, Krakow, Poland). The activation was carried out using carbon dioxide (technical CO_2_ 2.8, Linde Gaz Poland).

### 3.2. Preparation of Biocarbons

The initial material was separated into two distinct portions. One of the samples was subjected to pyrolysis at 500 °C for a period of 60 min. The process was conducted in a nitrogen atmosphere (170 mL/min). The resulting material was then activated with carbon dioxide (250 mL/min) at 800 °C for 45 min. The resulting adsorbent was designated P5D8 in this study. An alternative method for producing biochars involved direct activation of the precursor at 800 °C (D8). This process was conducted in a carbon dioxide atmosphere (250 mL/min) for 45 min. Carbonisation and activation were performed in a tube furnace.

### 3.3. Characterisation

The elemental composition of the precursor and biocarbons was determined using a Thermo Scientific FLASH 2000 Elemental Analyzer (ElementarAnalysensysteme GmbH, Langenselbold, Germany). The ash content of the activated carbons was evaluated by combusting the samples in a microwave muffle furnace at 815 °C for 60 min. The textural characterisation of the samples was conducted using N_2_ adsorption and desorption isotherms, measured at 77 K with a Quantachrome Autosorb Instrument (Boynton Beach, FL, USA). The specific surface area was calculated using the Brunauer–Emmett–Teller equation. Prior to measurements, the samples were degassed for 12 h at 300 °C. The total pore volume was estimated by considering the amount of nitrogen adsorbed at a relative pressure of approximately 0.99. The t-plot method was employed to ascertain the volume and surface area of the micropores. X-ray photoelectron spectroscopy (XPS) was conducted using an ultrahigh vacuum photoelectron spectrometer with a Phoibos150 NAP analyser (Specs). The analytical chamber operated in vacuum at a pressure close to 5 × 10^−9^ mbar, and the sample was irradiated with non-monochromatic Al Kα radiation (1486.6 eV). 

The Boehm titration method was used to determine the amount of acidic and basic surface functional groups on the biocarbons. This involved titrating with 0.1 mol/L NaOH or HCl, using a 1% methyl orange solution as an indicator. Each sample was tested in duplicate. To determine the pH_pzc_ of each sample, six beakers with 200 mL of 0.1 M NaCl were adjusted to pH values between 2 and 12 using 0.1 M NaOH and HCl solutions. Samples of 0.1 g of carbon were added to the NaCl solutions and shaken at 300 rpm for 24 h. The final pH of each solution was recorded, and the pHpzc was found by plotting the final pH against the initial pH and identifying the intercept on the *y*-axis [46].

### 3.4. Adsorption Experiments

The adsorption experiments were conducted under a range of conditions, including the concentration of methylene blue and rhodamine B (10–40 mg/L), pH level (3–12), adsorbent mass (0.005–0.03 g), shaking speed (100–300 rpm/min), contact time (6 h), and reaction temperature (298–328 K). The solutions of dyes were prepared using double-distilled water. In order to adjust the solution pH, 0.1 M NaOH and/or 0.1 M HCl was employed, as required. The measurements were performed twice, and the results obtained are the average of two independent determinations.

Portions of 0.02 g biocarbon were placed in flasks and flooded with 50 mL of a methylene blue/rhodamine B solution at concentrations ranging from 5 to 40 mg/L. The flasks were then subjected to a 24 h shaking process (200 rpm/min) at a temperature of 23 ± 1 °C. Following the completion of the shaking process, the solid contents were separated by means of centrifugation on a laboratory centrifuge (OHAUS, Parsippany, NJ, USA). The concentration of dyes remaining in the solution was determined spectrophotometrically using a Carry 100 Bio spectrophotometer (Agilent, Santa Clara, CA, USA). Rhodamine B exhibits a maximum absorption wavelength of 553 nm. Methylene blue exhibits absorption at λ_max_ = 664 nm. The amount of dyes adsorbed on the biocarbons was calculated from Equation (1):

(1) $$q_{e}=\frac{C_{0}-C_{e}}{m}\cdot V$$

where *C*_0_—initial dye concentration (mg/L); *C*_e_—equilibrium dye concentration (mg/L); *m*—the mass of sample (g); and *V*—volume of dye solution (L). The experimental adsorption studies were conducted on three occasions, and the results are presented with a standard deviation error.

The experimental data were subjected to a fitting process using the Langmuir (2), Freundlich (3), Temkin (4), and Dubinin–Radushkevich (5) isotherms [47].

(2) $$\frac{C_{e}}{q_{e}}=\frac{1}{K_{L}\cdot q_{max}}+\frac{C_{e}}{q_{max}}$$

(3) $$lnq_{e}=lnK_{F}+\frac{1}{n}lnC_{e}$$

(4) $$nq_{e}=BlnA_{T}+BlnC_{e}$$

(5) $$lnq_{e}=lnq_{max}-\beta \varepsilon^{2}$$

where *q_max_*—dye monolayer adsorption capacity (mg/g); *K_L_*—Langmuir adsorption constants (L/mg); *n*—Freundlich adsorption constants related to adsorption capacity; *K_F_*—Freundlich adsorption constants related to sorption intensity (mg/g (mg/L)^1/n^); *B*—constant related to the heat of adsorption (J/mol); *A_T_*—constant related to the maximum binding energy (L/mg); *ε*—the Polanyi potential; and *β*—constant which allows for the calculation of free energy *E* (kJ/mol) (6):

(6) $$E=\frac{1}{\sqrt{2\beta }}$$

Additionally, the experimental data were fitted to the pseudo-first-order (7), pseudo-second-order (8) and intraparticle diffusion (9) models, as outlined in reference [48].

(7) $$\frac{C_{e}}{q_{e}}=\frac{1}{K_{L}\cdot q_{max}}+\frac{C_{e}}{q_{max}}$$

(8) $$lnq_{e}=lnK_{F}+\frac{1}{n}lnC_{e}$$

(9) $$q_{t}=k_{IPD}t^{1/2}+C$$

where *q_t_*—sorption capacity of the dye adsorbed in time (mg/g); *k*_1_—the rate constant for the pseudo-first-order model (1/min); *k*_2_—the rate constant for the pseudo-second-order model (g/mg × min); *k_IPD_* (mg/g × min^1/2^)—the intraparticle rate constant; and *C* (mg/g)—the intercept. The value of C can be obtained from the plot of q_t_ versus *t*^1/2^.

Furthermore, sorption experiments were conducted on the prepared biocarbons at temperatures of 298, 318 and 338 K (10–12) in order to investigate the sorption behaviour of dyes on these materials [49].

(10) $$∆G^{0}=-RTlnK_{d}$$

(11) $$∆G^{0}=∆H^{0}-T∆S^{0}$$

(12) $$lnK_{d}=\frac{∆S^{0}}{R}-\frac{∆H^{0}}{RT}$$

where Δ*G*^0^—Gibbs free energy (kJ/mol); *R*—gas constant (J/mol × K); *T*—temperature (K); *K_d_*—thermodynamic equilibrium constant; Δ*H*^0^—enthalpy change (J/mol); and Δ*S*^0^—entropy change (J/mol × K).

### 3.5. Desorption Experiments

One gram of the D8 sample was mixed with 50 mL of a 30 mg/L methylene blue or rhodamine B solution for 8 h. After reaching adsorption equilibrium, the sample was drained and rinsed with distilled water to remove any remaining adsorbate. The adsorbents were then air-dried. Desorption was carried out using 0.1 M hydrochloric acid, 0.1 M sodium hydroxide solution and water as desorption solutions. The desorption process lasted for 12 h.

## 4. Conclusions

The cellulose fibres utilised in the production of infant nappy mats were employed as a precursor to biocarbons. The results of the physicochemical analysis demonstrated that the activation process exerts a significant influence on the surface area of the biomaterials in question. It has been demonstrated that the activation of carbon dioxide facilitates the formation of biochars with a slightly alkaline surface character. However, the primary focus of the present study was the adsorption process of methylene blue and rhodamine B on biocarbons. The adsorption of methylene blue and rhodamine B onto biocarbons was found to be in accordance with the Langmuir model. The kinetic studies indicated that the adsorption of both dyes on the activated carbons followed a pseudo-second-order model, implying a chemisorption mechanism between the adsorbent and the adsorbate. Thermodynamic analysis demonstrated that the adsorption process was endothermic and spontaneous on the tested adsorbents, as evidenced by an increase in the effectiveness of methylene blue/rhodamine B removal. Additionally, negative values of ∆G^0^ confirm the above statement.

The desorption tests indicated that the 0.1 M HCl solution was the most effective eluent for removing dyes.

The subsequent phase of the research will focus on modifying the activation methodology in order to produce biocarbon adsorbents with markedly enhanced textural characteristics. The planned experiments will investigate the effects of varying the activation times and temperatures. The optimisation of the process parameters for the synthesis of biocarbons will have a considerable impact on the cost of production. Moreover, adsorption tests will be performed on actual systems.

## Figures and Tables

**Table 1:** Textural parameters of the biocarbons.

Biocarbon	Surface Area (m^2^/g) *	Micropore Area (m^2^/g)	Total Pore Volume (cm^3^/g)	Micropore Volume (cm^3^/g)	Average Pore Diameter (nm)	Iodine Number (mg/g)	References
D8	450	221	0.38	0.09	3.36	440	
P5D8	384	274	0.25	0.16	2.57	328	
carbon from pistachio nut shell	877	841	0.64	0.54	2.92	-	[21]
carbon from hay	258	242	0.18	0.14	2.7	402	[29]
carbon from walnut shell	401	361	0.27	0.20	2.67	-	[30]

* error range between 2 and 5%.

**Table 2:** Acid–base properties of the biocarbons.

Biocarbon	Acidic Groups (mmol/g) *	Basic Groups (mmol/g) *	pH_pzc_ *
D8	0.48	1.06	9.8
P5D8	0.46	0.67	8.1

* arithmetic mean of the three determinations.

**Table 3:** Elemental analysis and ash content of the precursor and biocarbons (wt.%).

Sample	C^daf^	H^daf^	N^daf^	S^daf^	O^daf^ *	Ash
Precursor	42.13	7.17	0.54	0.16	50.00	0.1
D8	88.60	0.25	0.90	0.83	9.42	3.9
P5A8	92.11	0.28	0.78	0.51	6.32	3.2

daf–dry ash-free basis, *—by difference, method error ≤0.3%.

**Table 4:** Isotherm study of methylene blue and rhodamine B.

Isotherms	Parameters	Methylene Blue		Rhodamine B	
		D8	P5D8	D8	P5D8
	q_e_ (mg/g)	84	14	47	4
Langmuir	R^2^	0.999	0.996	0.999	0.979
	Adj R^2^	0.999	0.995	0.999	0.973
	q_max_	85	15	48	5
	K_L_ (L/mg)	0.007	0.089	0.003	0.133
Freundlich	R^2^	0.815	0.897	0.801	0.935
	Adj R^2^	0.778	0.871	0.801	0.926
	K_F_ (mg/g(L/mg)^1/n^)	41.869	4.449	28.836	1.479
	1/n	0.293	0.327	0.242	0.299
Temkin	R^2^	0.892	0.933	0.918	0.945
	Adj R^2^	0.871	0.917	0.897	0.932
	B (J/mol)	12.165	3.0337	5.687	0.854
	A_T_ (L/mg)	72.852	2.764	45.708	3.774
Dubinin-Radushkevich	R^2^	0.928	0.944	0.940	0.777
	Adj R^2^	0.914	0.930	0.936	0.720
	q_max_ (mg/g)	73.920	13.027	48.913	3.573
	E (kJ/mol)	3.622	5.623	5.263	8.559

**Table 5:** Methylene blue and rhodamine B maximum adsorption capacity of selected adsorbents.

Adsorbent/Precursor	Adsorbat	Maximum Adsorption Capacity [mg/g]	References
green tea (leaves)	methylene blue	85	[20]
walnut shell		68.9	[30]
wheat bran		255.01	[32]
rattan sawdust		294.14	[33]
D5		85	This study
P5D8		15	This study
kaolinite	rhodamine B	46.08	[34]
rice straw		73.47	[35]
bagasse pith		263.85	[36]
D8		48	This study
P5D8		5	This study

**Table 6:** Kinetic parameters for adsorption of methylene blue and rhodamine B.

Model	Parameters	Methylene Blue		Rhodamine B	
		D8	P5D8	D8	P5D8
	q_t_ (mg/g)	72	14	45	4
pseudo-first-order	q_e,cal_ (mg/g)	48	5	29	5
	R^2^	0.725	0.597	0.796	0.597
	AdjR^2^	0.686	0.539	0.767	0.539
	k_1_ (1/min)	2.764 × 10^−3^	3.984 × 10^−3^	7.047 × 10^−3^	3.984 × 10^−3^
pseudo-second-order	q_e,cal_ (mg/g)	58	13	48	3
	R^2^	0.999	0.999	0.994	0.999
	AdjR^2^	0.999	0.999	0.994	0.999
	k_2_ (g/mg × min)	7.530 × 10^−4^	1.001 × 10^−2^	4.930 × 10^−4^	3.948 × 10^−2^
intraparticle diffusion model	C (mg/g)	12.95	5.72	5.36	1.37
	R^2^	0.801	0.595	0.816	0.583
	AdjR^2^	0.772	0.539	0.788	0.523
	k_IPD_ (mg/g × min)^1/2^	2.435	0.410	2.194	0.087

**Table 7:** Thermodynamic parameters of the adsorption of methylene blue/rhodamine B on biocarbons.

Biocarbon	Dye	q_e_ (mg/g)	Temperature (K)	∆G^0^ (kJ/mol)	∆H^0^ (kJ/mol)	∆S^0^ (J/mol K)
D8	methylene blue	67	298	−8.0	62.6	235.3
		69	308	−9.5		
		71	318	−11.3		
		72	328	−15.3		
P5D8		11	298	−1.3	10.3	27.7
		12	308	−1.4		
		14	318	−1.9		
		15	328	−2.0		
D8	rhodamine B	42	298	−4.1	10.7	49.8
		45	308	−4.8		
		45	318	−5.2		
		47	328	−5.6		
P5D8		4	298	−4.2	9.1	13.9
		4	308	−4.6		
		4	318	−4.7		
		5	328	−4.8		

**Table 8:** Regeneration of D8 sample by different eluents (%).

Sample	Dye	HCl	H_2_O	NaOH
D8	methylene blue	79	61	33
	rhodamine B	76	55	23

## Data Availability

Data are contained within the article.
